# Bronchoscopy in the post-acute phase of COVID-19: an observational study

**DOI:** 10.1186/s12890-023-02477-6

**Published:** 2023-05-22

**Authors:** Michele Mondoni, Rocco Francesco Rinaldo, Jacopo Cefalo, Laura Saderi, Beatrice Vigo, Paolo Carlucci, Claudio Tirelli, Umberto Cariboni, Pierachille Santus, Stefano Centanni, Giovanni Sotgiu

**Affiliations:** 1 Respiratory Unit, Department of Health Sciences, ASST Santi Paolo e Carlo, Università degli Studi di Milano, Via A. Di Rudinì n.8, Milan, 20142 Italy; 2 Clinical Epidemiology and Medical Statistics Unit, Dept of Medical, Surgical and Experimental Sciences, University of Sassari, Sassari, Italy; 3 Division of Thoracic Surgery, IRCCS Humanitas Research Hospital, Rozzano, Milan, Italy; 4 Division of Respiratory Diseases, Department of Biomedical and Clinical Sciences (DIBIC), ASST Fatebenefratelli-Sacco, Università degli Studi di Milano, Ospedale Luigi Sacco, Polo Universitario, Milano, Italy

**Keywords:** Tracheostomy; Bronchoscopy, Tracheal stenosis, Long COVID, COVID-19 sequelae, Lung cancer

## Abstract

**Background:**

Bronchoscopy is a useful technique adopted in the management of patients with COVID-19. 10–40% of COVID-19 survivors experience persistent symptoms. A comprehensive description of the utility and safety of bronchoscopy in the management of patients with COVID-19 sequelae is lacking. The aim of the study was to evaluate the role of bronchoscopy in patients with suspected post-acute sequelae of COVID-19.

**Methods:**

An observational, retrospective study was carried out in Italy. Patients requiring bronchoscopy for suspected COVID-19 sequelae were enrolled.

**Results:**

45 (21, 46.7%, female) patients were recruited. Bronchoscopy was more frequently indicated for patients with a previous critical disease. The most frequent indications were tracheal complications, mostly performed in patients who were hospitalized during the acute phase than treated at home (14, 48.3% VS. 1, 6.3%; p-value: 0.007) and persistent parenchymal infiltrates, more frequent in those treated at home (9, 56.3% VS. 5, 17.2%; p-value: 0.008). 3 (6.6%) patients after the first bronchoscopy required higher oxygen flow. Four patients were diagnosed with lung cancer.

**Conclusion:**

Bronchoscopy is a useful and safe technique in patients with suspected post-acute sequelae of COVID-19. The severity of acute disease plays a role in the rate and indications of bronchoscopy. Endoscopic procedures were mostly performed for tracheal complications in critical, hospitalized patients and for persistent lung parenchymal infiltrates in mild-moderate infections treated at home.

**Supplementary Information:**

The online version contains supplementary material available at 10.1186/s12890-023-02477-6.

## Introduction

Bronchoscopy is a useful and safe technique in the management of patients with Coronavirus Disease 2019 (COVID-19) [1–3].

It shows a good sensitivity to diagnose severe acute respiratory syndrome coronavirus 2 (SARS-CoV-2) in patients with a negative nasopharyngeal swab and a clinical and radiological suspicion of COVID-19 pneumonia [1–3]. It plays a key role to rule-out other viral pneumonias and to detect bacterial or fungal co-infections. Urgent/life-saving therapeutic bronchoscopies can be successfully performed in critical patients with COVID-19 in case of obstructive atelectasis, hemoptysis, or tracheostomy complications [1–3].

The majority of COVID-19 survivors shows a full recovery [4, 5]. Although the exact prevalence of SARS-CoV-2 long-term sequelae is still unknown, it is estimated that approximately 10–40% experience persistent symptoms for weeks to months following the acute infection [4–7]. For patients with suspected/proven pulmonary sequelae, international guidelines recommend radiological and a functional assessment, but no specific indications on bronchoscopy are provided [4].

A recent study focused on the utility of endoscopic techniques in the management of twenty-three patients with post-intubation tracheal stenosis and/or tracheoesophageal fistulas [8].

To our knowledge, a comprehensive description of the utility and safety of bronchoscopy in the management of patients with suspected COVID-19 sequelae is lacking.

The aim of the study was to evaluate the role of bronchoscopy in patients with persistent symptoms and suspected post-acute sequelae of COVID-19. Furthermore, we estimated the impact of acute infection on the number of bronchoscopies, the type of endoscopic procedures and their safety profile.

## Materials and methods

### Study design

An observational, retrospective, monocentre study was carried out in Italy, after the approval of the local ethical committee. Written informed consent was signed by recruited patients.

### Patients and interventions

From June 2020 to August 2022 adult (i.e., ≥ 18 years old) consecutive patients with virologically confirmed previous SARS-CoV-2 infection and requiring bronchoscopy for suspected COVID-19 sequelae were enrolled. According to World Health Organization (WHO) consensus statement on post-COVID-19 condition, patients were deemed suitable for enrollment at least 3 months from the onset of COVID-19 with new or persistent or fluctuant or relapsing symptoms lasting for at least 2 months [5].

Exclusion criteria were the following: bronchoscopies performed in patients with previous COVID-19 < 3 months from the onset of disease, procedures not specifically related to previous acute viral infection (i.e. performed for new symptoms and/or new radiological abnormalities after clinical and radiological recovery), and/or refusal to sign the informed consent.

At the time of enrollment patients had a negative SARS-CoV-2 nasopharyngeal swab and, at least, one chest computed tomography (CT) scan. Bronchoscopic procedures were performed under conscious or deep sedation (flexible bronchoscopy) or general anesthesia (rigid bronchoscopy).

Demographic, clinical, and endoscopic characteristics at baseline and during previous acute infection were recorded.

### Outcomes

The primary outcome was to assess the indications of bronchoscopy in patients with suspected post COVID-19 sequelae.

Furthermore, the number of bronchoscopies, the endoscopic techniques, the rate of adverse events, the final diagnosis, and the clinical outcomes were recorded.

Finally, the relationship between the number of bronchoscopies, hospital admission during the acute infection, and the severity of acute disease according WHO classification was studied [9].

### Statistical analysis

An ad hoc electronic form was created to collect qualitative and quantitative variables. Qualitative covariates were summarized with absolute and relative (percentage)frequencies. Means (standard deviations, SD) or medians (interquartile ranges, IQR) were used to describe quantitative variables in case of parametric and non-parametric distributions, respectively. Chi-square or Fisher’s exact test was performed to detect any statistical differences in the comparison of the qualitative variables, whereas between-group comparisons of quantitative variables were performed with Student’s t-test and Mann-Whitney test for parametric and non-parametric variables, respectively. A p-value less than 0.05 was considered statistically significant. The statistical software Stata 17 (StataCorp, TX) was used for all statistical computations.

## Results

Overall, 202 patients with previous COVID-19 underwent bronchoscopy in the post-acute phase. A total of 45 (21, 46.7% female) patients (mean (SD) age: 61.4 (11.9) years) met inclusion criteria (Table 1).

**Table 1:** Demographic and clinical characteristics of the cohort

Mean (SD) age, years:		61.4 (11.9)
Female, n (%)		21 (46.7)
Ethnicity, n (%)	Caucasian	38 (84.4)
	Hispanic	5 (11.1)
	Asian	1 (2.2)
	African	1 (2.2)
Mean (SD) BMI, kg/m^2^		26.3 (5.2)
Smoking history, n (%)	Never smoker	31 (68.9)
	Current smoker	2 (4.4)
	Former smoker	12 (26.7)
Mean (SD) pack/years		24.9 (16.6)
Comorbidities, n (%)		
Chronic respiratory diseases	COPD	4 (8.9)
	ILD	3 (6.7)
	Asthma	2 (4.4)
	Bronchiectasis	1 (2.2)
	OSAS	1 (2.2)
Systemic arterial hypertension		16 (35.6)
Ischemic cardiomyopathy		3 (6.7)
Diabetes		11 (24.4)
Chronic kidney disease		3 (6.7)
Haematological malignancy		2 (4.4)

SD: standard deviation; BMI: body mass index; COPD: chronic obstructive pulmonary disease; ILD: interstitial lung disease; OSAS: obstructive sleep apnea syndrome

30 (66.6%) patients were admitted to the hospital during acute infection and the majority had a critical acute disease. 17 required tracheal intubation for a mean (SD) of 10.2 (4.8) days: they required a mechanical ventilation and 16 (94.1%) underwent tracheostomy (Table 2).

**Table 2:** Characteristics of the acute COVID-19 in patients undergoing bronchoscopy in the post-acute phase

Median (IQR) hospital length of stay, days (n = 30)		53.5 (32–81)
Severity of acute infection (WHO definition)	Mild	13 (29.6)
	Moderate	6 (13.6)
	Severe	6 (13.6)
	Critical	19 (43.2)
Max ventilatory support	Room air	11 (24.4)
	Supplemental oxygen	8 (17.8)
	HFNC	3 (6.7)
	CPAP	4 (8.9)
	NIV	2 (4.4)
	Tracheal intubation + invasive mechanical ventilation	17 (37.8)
Type of bronchoscope	Flexible	16 (69.6)
	Flexible bronchoscope through tracheal tube	7 (30.4)
Indications	Suspected COVID-19	3 (13.0)
	Haemoptysis	2 (8.7)
	Suspected tracheal stenosis	3 (13.0)
	Obstructive atelectasis	3 (13.0)
	Management of tracheostomy complication (granuloma)	1 (4.3)
	Decannulation	11 (47.8)

WHO: World Health Organization; HFNC: high flow nasal cannula; CPAP: continuous positive airway pressure; NIV: non-invasive ventilation; COVID-19: coronavirus disease- 2019; IQR: interquartile range

In 17/45 (37.8%) recruited patients who underwent bronchoscopy in the post-acute phase, an endoscopic examination was also performed during the acute phase of disease. Overall, 23 bronchoscopies were performed during the acute disease in 17 patients: 11 (64.7%) underwent one bronchoscopy, 5 (29.4%) and 1 (5.8%) patient underwent two and three endoscopic evaluations, respectively.

The median (IQR) time between symptoms onset of COVID-19 and the first bronchoscopy in the post-acute phase was 4 (3–6) months.

The most prevalent symptoms at the time of the first bronchoscopy in the post-acute phase were dyspnoea (18, 40%) and cough (7, 15.5%) (Table S1).

A total of 58 bronchoscopies were performed in the post-acute phase. 55 (94.8%) with the flexible bronchoscope and 3 (5.2%) with the rigid scope. 11 (24.4%) patients underwent two procedures and 2 (4.4%) patients required three bronchoscopies.

Overall, in the post-acute phase bronchoscopies were more frequently indicated for patients with a previous critical acute disease. The need for one bronchoscopy was more frequent in patients with a mild acute disease whereas a second and a third bronchoscopy were more likely performed in patients with critical COVID-19 (Table 3).

**Table 3:** Number of bronchoscopies during the post-acute phase related to severity of acute disease

		Severity of acute infection (WHO definition)				
		Mild (n = 12)	Moderate (n = 6)	Severe (n = 6)	Critical (n = 19)	p-value
Median (IQR) no. of bronchoscopies during post-acute phase		1 (1–1)	1 (1–1)	1 (1–2)	1 (1–2)	0.02*
No. of bronchoscopies during the post-acute phase	1	12 (100.0)	6 (100.0)	4 (66.7)	10 (52.6)	0.01**
	2	0 (0.0)	0 (0.0)	1 (16.7)	8 (42.1)	0.02***
	3	0 (0.0)	0 (0.0)	1 (16.7)	1 (5.3)	0.57

*Mild VS. Critical p-value = 0.01

**Mild VS. Severe p-value = 0.03; Mild VS. Critical p-value = 0.005; Moderate VS. Critical p-value = 0.04

*** Mild VS. Critical p-value = 0.01; Mild VS. Severe p-value = 0.04

WHO: World Health Organization

The most frequent indications were tracheal complications and persistent parenchymal infiltrates (Table 4). The former was more frequent in patients who were admitted to the hospital during the acute phase than treated at home (14, 48.3% VS. 1, 6.3%; p-value: 0.007), the latter in patients treated at home (9, 56.3% VS. 5, 17.2%; p-value: 0.008) (Table 4).

**Table 4:** Indications for bronchoscopy in post-acute phase according to hospital admission during the acute infection

	Indication for bronchoscopy	Non-Hospitalized in the acute phase (n = 16)	Hospitalized in the acute phase (n = 29)	*p*-value
*Bronchoscopy 1*	Suspected tracheal stenosis	1 (6.3)	4 (13.8)	0.44
	Therapeutic management of tracheal stenosis	0 (0.0)	2 (6.9)	0.28
	Follow-up of a known tracheal stenosis/tracheostomy complication (e.g., granuloma)	0 (0.0)	8 (27.6)	0.02
	Tracheal complications (all previous together)	1 (6.3)	14 (48.3)	0.007
	Assistance during decannulation	0 (0.0)	1 (6.3)	0.17
	Haemoptysis	1 (6.3)	0 (0.0)	0.17
	Persistent parenchymal infiltrates at chest imaging	9 (56.3)	5 (17.2)	0.008
	Suspected late infection	2 (12.5)	6 (20.7)	0.49
	Obstructive atelectasis	1 (6.3)	2 (6.9)	0.94
	Presence of lymphadenopathies at CT scan	1 (6.3)	1 (3.5)	0.66
*Bronchoscopy 2*	Therapeutic management of tracheal stenosis	0/1 (0.0)	2/10 (20.0)	
	Follow-up of a known tracheal stenosis/tracheostomy complication (e.g., granuloma)	1/1 (100.0)	4/10 (40.0)	
	Persistent parenchymal infiltrates at chest imaging	0/1 (0.0)	2/10 (20.0)	
	Obstructive atelectasis	0/1 (0.0)	1/10 (10.0)	
	Occurrence of lymphadenopathies at CT scan	0/1 (0.0)	1/10 (10.0)	
*Bronchoscopy 3*	Follow-up of a known tracheal stenosis/tracheostomy complication (e.g., granuloma)	-	1/2 (50.0)	
	Suspected late infection	-	1/2 (50.0)	

CT: computed tomography

No differences were detected between smokers and non-smokers in the indications for the first bronchoscopy overall (p-value: 0.24) and in those related to tracheal complications (p-value: 0.21).

Bronchial washing and bronchoalveolar lavage (BAL) were the most employed sampling techniques: they were used in 5 (11.1%) and 18 (40%) patients during the first bronchoscopy, in 5 (45.5%) and 2 (18.2%) during the second and both in 1 (50%) during the third bronchoscopy, respectively (Table S2).

Therapeutic bronchoscopy was performed in 7 (15.5%) patients; in 2 (28.6%) patients tracheal granulomas were treated with bronchoscopic argon plasma coagulation (APC); in 2 (28.6%) tracheal stenosis were treated with laser therapy, and in 1 (14.3%) with APC. Other 2 (28.6%) patients had bronchoscopic mucus plug removal. 4 (57.1%) patients fully recovered, whereas 2 (28.6%) with obstructive atelectasis needed a second procedure, and 1 (14.3%) case of tracheal stenosis relapsed and was definitely treated with surgical tracheal anastomosis.

Overall, 3 (6.6%) patients after the first bronchoscopy required subsequent higher oxygen flow; no other complications were recorded.

5 (66.7%) patients, who underwent bronchoscopy for persistent parenchymal infiltrates (one with concomitant lymphadenopathies at CT scan), did not receive a specific final diagnosis after the first bronchoscopy. After a second one two patients were finally diagnosed with sarcoidosis (with endoscopic ultrasound with bronchoscope fine needle aspiration, EUS-B-FNA of a mediastinal lymph node) and organizing pneumonia (with transbronchial biopsy). In the remaining three patients persistent lung infiltrates were diagnosed as non-specific residual post-COVID-19 fibrosis.

Four patients were diagnosed with lung cancer (adenocarcinomas) (Table 5). All had mild-moderate COVID-19 and were treated at home. Only one of them had a chest X-ray during acute infection, showing a lung infiltrate. In the post-acute phase, chest CT scan showed lobar atelectasis in one patient, diffuse ground glass opacities with segmental alveolar infiltrate in another patient, and lobar alveolar infiltrates with septal thickening suggestive of lymphangitic carcinomatosis in other two patients.

**Table 5:** Final diagnosis/outcome after all the endoscopic examinations related to the indication for the first bronchoscopy

Patients number	Indication for the first bronchoscopy	Final diagnosis/endoscopic outcome after all the bronchoscopies
5	Suspected tracheal stenosis	5 tracheal stenosis (1 treated with laser with relapse)
2	Therapeutic management of tracheal stenosis	2 (1 treated with laser; 1 with APC)
8	Follow-up of a known tracheal stenosis/tracheostomy complication	4 granulomas (2 removed with APC), 4 stenosis
2	Assistance during decannulation	2 patients decannulated
1	Haemoptysis	1 sarcoidosis
14	Parenchymal infiltrates at chest imaging persistent after the resolution of acute disease	3 Lung cancer (adenocarcinoma) 4 LRTI 4 ILD (2 NSIP, 2 organizing pneumonia) 3 Residual non-specific post-COVID19 fibrosis
8	Clinical-radiological suspicion of late infections	6 LRTI 1 ILD (organizing pneumonia) 1 Tracheal stenosis and LRTI
3	Atelectasis	1 Lung cancer (adenocarcinoma) 2 Obstructive atelectasis (mucus plug removal)
2	Presence of lymphadenopathies at CT scan	2 sarcoidosis

APC: Argon Plasma Coagulation; LRTI: lower respiratory tract infection: ILD: interstitial lung disease; NSIP: non-specific interstitial pneumonia; COVID-19: coronavirus disease – 2019; CT: computed tomography

5 (11.1%) patients (all hospitalized during acute infection) were diagnosed with a lower respiratory tract infection caused by *Candida albicans* and *Cytomegalovirus* in one patient, *Pseudomonas aeruginosa* and methicillin-sensitive *Staphylococcus aureus* in two patients, respectively.

## Discussion

This is the first study aimed at evaluating the utility and safety of bronchoscopy in the post-acute phase of COVID-19. Tracheal complications and persistent large parenchymal infiltrates were the most frequent indications for endoscopy in symptomatic patients who were hospitalized and non-hospitalized during the acute disease, respectively.

Previous studies showed that the severity of acute COVID-19 may significantly impact on the extent of lung abnormalities at CT scan [10–12] but does not influence lung function and exercise capacity three months after the discharge [13].

Although patients with previous mild disease underwent bronchoscopy, those with acute critical disease required a more elevated number of endoscopic procedures in the post-acute phase, mostly related to tracheal complications.

The prevalence of post-intubation tracheal complications in patients with COVID-19 is still unknown [8]. Other Authors found an increased incidence of tracheal disease, which could either be attributed to the increase of intubated patients in emergency, prolonged intubation, disease characteristics (e.g. high viral replication in the tracheal epithelium) and/or patients’ comorbidities [8, 14–16]. In our study bronchoscopy was successfully employed as a therapeutic option, mostly in post-intubation/tracheostomy complications, confirming the findings of a study performed in Greece [8].

Notably, when compared with indications for bronchoscopies during the acute infection, those related to the management of tracheal complications were significantly more prevalent in the post-acute phase [1–3, 8].

Few cases of incidental or concomitant diagnosis of lung cancer during acute COVID-19 or in the post-acute phase were described in the literature [17, 18].

In our study four patients with previous acute mild-moderate disease with persistent respiratory symptoms were diagnosed with lung adenocarcinomas. Patients with non-resolving symptoms after a mild acute infection should undergo chest imaging to rule out other potentially life-threatening diseases.

Some patients with persistent symptoms and residual large parenchymal infiltrates were diagnosed with interstitial lung disease (ILD) (i.e. sarcoidosis, organizing pneumonias (OP) and non-specific interstitial pneumonia (NSIP). Although post-COVID-19 OP and NSIP were previously described [10, 19, 20], it is unclear whether some ILD diagnosed in the post-acute phase were triggered by or were pre-existing the acute infection.

We first assessed the utility of bronchoscopy in the diagnosis of lower respiratory tract infections following acute COVID-19; microorganisms were diagnosed using bronchial washing and BAL, which are the most useful sampling techniques in suspected respiratory tract infections [21]. Prolonged hospitalization and immunosuppression during acute infection (i.e., corticosteroids treatment) may increase the risk of concomitant or late infections [22–24].

This study has some limitations related to the small sample size, its observational retrospective nature, the monocenter design, and the imbalanced recruitment of groups with different disease severity.

## Conclusions

In conclusion, bronchoscopy is a useful and safe technique in patients with suspected post-acute sequelae of COVID-19. The severity of acute disease can play a role in the rate and indications of bronchoscopy. Endoscopic procedures were mostly performed for tracheal complications in critical, hospitalized patients and for persistent lung parenchymal infiltrates in mild-moderate infections treated at home.

Patients with persistent symptoms after the resolution of acute infection should immediately undergo a clinical and imaging re-evaluation. Bronchoscopy may be crucial to manage tracheal complications and obstructive atelectasis and in the diagnostic work-up of persistent lung infiltrates potentially related to other serious lung diseases.

## Electronic supplementary material

Below is the link to the electronic supplementary material.

Supplementary Material 1

## Data Availability

all data generated or analysed during this study are included in this article. Further enquiries can be directed to the corresponding author.

## Abbreviations

COVID-19: Coronavirus Disease 2019
SARS-CoV-2: severe acute respiratory syndrome coronavirus 2
WHO: World Health Organization
SD: standard deviation
IQR: interquartile ranges
CT: computed tomography
BAL: bronchoalveolar lavage
APC: argon plasma coagulation
EUS-B-FNA: endoscopic ultrasound with bronchoscope fine needle aspiration

## References

[1] Mondoni M, Papa GFS, Rinaldo R, Faverio P, Marruchella A, D’Arcangelo F (2020). Utility and safety of bronchoscopy during the SARS-CoV-2 outbreak in Italy: a retrospective, multicentre study. Eur Respir J.

[2] Arenas-De Larriva M, Martín-DeLeon R, Urrutia Royo B, Fernández-Navamuel I, Gimenez Velando A, Nuñez García L et al. The role of bronchoscopy in patients with SARS-CoV-2 pneumonia. ERJ open Res. 2021;7. 10.1183/23120541.00165-2021 PMC8183029 34258257

[3] Saha BK, Saha S, Chong WH, Beegle S, Indications (2022). Clinical utility, and Safety of Bronchoscopy in COVID-19. Respir Care.

[4] Antoniou KM, Vasarmidi E, Russell A-M, Andrejak C, Crestani B, Delcroix M et al. European respiratory society statement on long COVID follow-up. Eur Respir J. 2022;60. 10.1183/13993003.02174-2021 PMC9349784 35144991

[5] A clinical case. definition of post COVID-19 condition by a Delphi consensus, 6 October 2021. World Health Organization 2021. WHO reference number: WHO/2019-nCoV/Post_COVID-19_condition/Clinical_case_definition/2021.1.

[6] Bellan M, Baricich A, Patrucco F, Zeppegno P, Gramaglia C, Balbo PE (2021). Long-term sequelae are highly prevalent one year after hospitalization for severe COVID-19. Sci Rep.

[7] Rinaldo RF, Mondoni M, Parazzini EM, Pitari F, Brambilla E, Luraschi S (2021). Deconditioning as main mechanism of impaired exercise response in COVID-19 survivors. Eur Respir J.

[8] Stratakos G, Anagnostopoulos N, Alsaggaf R, Koukaki E, Bakiri K, Emmanouil P et al. COVID-19 patients presenting with Post-Intubation Upper Airway Complications: a parallel epidemic? J Clin Med. 2022;11. 10.3390/jcm11061719 PMC8948992 35330044

[9] Clinical management of COVID-19. : living guideline, 15 September 2022. Geneva: World Health Organization; 2022 (WHO/2019-nCoV/Clinical/2022.2). Licence: CC BY-NC-SA 3.0 IGO. 35917394

[10] Solomon JJ, Heyman B, Ko JP, Condos R, Lynch DA (2021). CT of Post-Acute Lung Complications of COVID-19. Radiology.

[11] Huang Y, Tan C, Wu J, Chen M, Wang Z, Luo L (2020). Impact of coronavirus disease 2019 on pulmonary function in early convalescence phase. Respir Res.

[12] Morin L, Savale L, Pham T, Colle R, Figueiredo S, Harrois A (2021). Four-Month Clinical Status of a cohort of patients after hospitalization for COVID-19. JAMA.

[13] Rinaldo RF, Mondoni M, Parazzini EM, Baccelli A, Pitari F, Brambilla E (2021). Severity does not impact on exercise capacity in COVID-19 survivors. Respir Med.

[14] Scholfield DW, Warner E, Ahmed J, Ghufoor K (2021). Subglottic and tracheal stenosis associated with coronavirus disease 2019. J Laryngol Otol.

[15] Bradley BT, Maioli H, Johnston R, Chaudhry I, Fink SL, Xu H (2020). Histopathology and ultrastructural findings of fatal COVID-19 infections in Washington State: a case series. Lancet (London England).

[16] Fiacchini G, Tricò D, Ribechini A, Forfori F, Brogi E, Lucchi M (2021). Evaluation of the incidence and potential mechanisms of Tracheal Complications in patients with COVID-19. JAMA Otolaryngol Head Neck Surg.

[17] Zhang Y, Li J, Li Z-K, Yang X, Bai J, Liu L (2021). Impact of Coronavirus Disease 2019 on clinical characteristics in patients with Lung Cancer: a large single-centre Retrospective Study. Front Oncol.

[18] González J, Zuil M, Benítez ID, de Gonzalo-Calvo D, Aguilar M, Santisteve S (2022). One year overview and Follow-Up in a Post-COVID Consultation of critically ill patients. Front Med.

[19] Besutti G, Monelli F, Schirò S, Milone F, Ottone M, Spaggiari L (2022). Follow-Up CT patterns of residual lung abnormalities in severe COVID-19 pneumonia survivors: a Multicenter Retrospective Study. Tomogr (Ann Arbor Mich).

[20] Wang Y, Jin C, Wu CC, Zhao H, Liang T, Liu Z (2020). Organizing pneumonia of COVID-19: time-dependent evolution and outcome in CT findings. PLoS ONE.

[21] Mondoni M, Rinaldo RF, Carlucci P, Terraneo S, Saderi L, Centanni S (2022). Bronchoscopic sampling techniques in the era of technological bronchoscopy. Pulmonology.

[22] Ceballos ME, Nuñez C, Uribe J, Vera MM, Castro R, García P (2022). Secondary respiratory early and late infections in mechanically ventilated patients with COVID-19. BMC Infect Dis.

[23] Cohen R, Finn T, Babushkin F, Geller K, Alexander H, Shapiro M (2022). High rate of bacterial respiratory tract co-infections upon admission amongst moderate to severe COVID-19 patients. Infect Dis (London England).

[24] Hughes S, Troise O, Donaldson H, Mughal N, Moore LSP (2020). Bacterial and fungal coinfection among hospitalized patients with COVID-19: a retrospective cohort study in a UK secondary-care setting. Clin Microbiol Infect Off Publ Eur Soc Clin Microbiol Infect Dis.

