# Supplementary material for: Bronchoscopy in the post-acute phase of COVID-19: an observational study
Source: BMC Pulm Med. 2023 May 22;23:178. doi: 10.1186/s12890-023-02477-6 (PMC10202063; doi:10.1186/s12890-023-02477-6)
Supplement: Supplementary file 1 — Supplementary Material 1 [file 12890_2023_2477_MOESM1_ESM.docx]

**Supplementary material**

Table S1. Symptoms at the time of the first bronchoscopy in the post-acute phase

| Dyspnoea | 18 (40) |
| --- | --- |
| Cough | 7 (15.5) |
| Fatigue | 2 (4.4) |
| Haemoptysis | 1 (2.2) |
| Wheezing | 1 (2.2) |
| Fever | 2 (4.4) |
| Dyspnoea and cough | 4 (8.8) |
| Dyspnoea and fatigue | 1 (2.2) |
| Dyspnoea and wheezing | 4 (8.8) |
| Dyspnoea and fever | 1 (2.2) |
| Cough and fever | 3 (6.6) |
| Cough and fatigue | 1 (2.2) |

Table S2. Sampling techniques employed during bronchoscopy in the post-acute phase.

| ***Bronchoscopy 1 (n= 45 patients)*** | |
| --- | --- |
| *Bronchial washing* | 5 (11.1) |
| *BAL* | 18 (40.0) |
| *Bronchial biopsy* | 2 (4.4) |
| *Transbronchial biopsy* | 3 (6.7) |
| *EBUS-TBNA* | 4 (8.9) |
| *EUS-B-FNA* | 1 (2.2) |
| ***Bronchoscopy 2 (n=11 patients)*** | |
| *Bronchial washing* | 5 (45.5) |
| *BAL* | 2 (18.2) |
| *Bronchial biopsy* | 1 (9.1) |
| *EUS-TBNA* | 2 (18.2) |
| *EUS-B-FNA* | 1 (9.1) |
| ***Bronchoscopy 3 (n=2 patients)*** | |
| *Bronchial washing* | 1 (50.0) |
| *BAL* | 1 (50.0) |

BAL: bronchoalveolar lavage; EBUS-TBNA: endobronchial ultrasound transbronchial needle aspiration; EUS-B-FNA: endoscopic ultrasound with bronchoscope fine needle aspiration
